# Transcriptome and metabolome analysis of liver and kidneys of rats chronically fed NK603 Roundup-tolerant genetically modified maize

**DOI:** 10.1186/s12302-017-0105-1

**Published:** 2017-02-07

**Authors:** Robin Mesnage, Matthew Arno, Gilles-Eric Séralini, Michael N. Antoniou

**Affiliations:** 1 Department of Medical and Molecular Genetics, Gene Expression and Therapy Group, Faculty of Life Sciences & Medicine, King’s College London, 8th Floor, Tower Wing, Guy’s Hospital, Great Maze Pond, London, SE1 9RT UK; 2 Genomics Centre, King’s College London, Waterloo Campus, 150 Stamford Street, London, SE1 9NH UK; 3 Institute of Biology, EA 2608 and Risk Pole, MRSH-CNRS, University of Caen, Esplanade de la Paix, 14032 Caen Cedex, France

**Keywords:** Roundup, Glyphosate, GMO, Transcriptome, Metabolome, Toxicity

## Abstract

**Background:**

A previous 2-year rat feeding trial assessing potential toxicity of NK603 Roundup-tolerant genetically modified maize revealed blood and urine biochemical changes indicative of liver and kidney pathology. In an effort to obtain deeper insight into these findings, molecular profiling of the liver and kidneys from the same animals was undertaken.

**Results:**

Transcriptomics showed no segregation of NK603 maize and control feed groups with false discovery rates ranging from 43 to 83% at a cut-off *p* value of 1%. Changes in gene expression were not reflective of liver and kidney toxic effects. Metabolomics identified 692 and 673 metabolites in kidney and liver, respectively. None of the statistically significant disturbances detected (12–56 for different test groups) survived a false discovery rate analysis. Differences in these metabolites between individual animals within a group were greater than the effect of test diets, which prevents a definitive conclusion on either pathology or safety.

**Conclusions:**

Even if the biological relevance of the statistical differences presented in this study is unclear, our results are made available for scrutiny by the scientific community and for comparison in future studies investigating potential toxicological properties of the NK603 corn.

**Electronic supplementary material:**

The online version of this article (doi:10.1186/s12302-017-0105-1) contains supplementary material, which is available to authorized users.

## Background

The application of genetic modification (recombinant DNA, transgenic) technologies in agricultural practice has been advocated as an important advance in recent decades [1]. As they are made to meet the food needs of the entire human and most farm animal populations, the safety of plant products derived from this type of biotechnology is an important consideration and has been a matter of great debate. While advocates put forward evidences, which they suggest prove their safety [2, 3], others provide evidence-based arguments that show a lack of a scientific consensus on the safety of genetically modified (GM) foodstuffs [4].

Part of the concern raised by these GM crops rests on the fact that to date the vast majority are engineered to either tolerate application of a herbicide or produce a new systemic insecticide or both, which can result in elevated levels of these substances in food and feed [5]. In 2014, insecticide production and herbicide tolerance traits were deployed singly or in combination (“stacked”) in all agricultural GM commodity crops namely maize, soybeans, cotton and canola [5]. These GM crops were collectively planted globally on 181 million hectares in 28 countries, which represents approximately 8% of total global cropland, although cultivation was concentrated (>90%) in just 6 nations [5].

Approximately, 80% of all GM crops have been designed to tolerate application of glyphosate-based herbicides (GBHs), with Roundup being the major commercial brand. GBHs were first sold in 1974 and since then their use has increased 100-fold, with the vast majority (two-thirds) of the use having taken place in the last 10 years; that is, since the introduction of GBH-tolerant GM crops [6]. As a result, GBH-tolerant GM crops accumulate residues of these herbicides during cultivation [7] potentially increasing the daily intake of the consumer. In addition, the quantity of GM crop ingredients in laboratory rodent feed correlates with their content in GBH residues [8]. The safety of GBH residue consumption is highly controversial as some studies have demonstrated toxic effects in laboratory animals [9, 10], farm animals [11], as well as possible carcinogenic effects in humans [12]. This has led some commentators to suggest that there exists a gap between criteria used by regulators for market approval of GBHs and the advancing scientific evidence base questioning the safety of this class of herbicide [13].

Other concerns regarding sources of potential GM food toxicity stem from the molecular biological outcomes of the transformation process. In addition to bringing about a novel combination of gene functions, the GM transformation process as used to generate currently commercialized crops results in random transgene insertion with a risk of insertional mutagenesis. Furthermore, the plant tissue culture phase employed in the vast majority of GM transformation procedures is known to bring about large numbers of random genome-wide mutations and even chromosomal rearrangements, a phenomenon known as “somaclonal variation” [14–16]. As a result, the GM transformation process as a whole may inadvertently activate, inactivate, under- or overexpress one or more host genes. For example, in Roundup-tolerant GM soybeans, it has been found that read-through transcripts into sequences present downstream of the transgene integration site resulted in four different RNA variants, which might code for unknown fusion proteins [17]. It is therefore perhaps not surprising to find that studies using molecular profiling (proteomic, metabolomic) techniques have found significant compositional differences between the GM line and non-GM isogenic parent. These methods have shown marked protein and metabolite profile differences induced by the GM transformation process in numerous crops such as maize [18–20], potatoes [21], rice [22, 23] and cotton [24]. These types of protein and metabolic changes can not only modify crop performance [25, 26] but could also change the nutritional and toxicological profile of the transgenic plant [27].

In order to evaluate the safety of GM food consumption, including for the purposes of obtaining market approval, several studies consisting of 90-day feeding trials in rats have been conducted, which analysed groups of 10 animals. These investigations have frequently resulted in statistically significant differences in parameters reflective of liver and kidney biochemistry, but with interpretation of their biological significance being a point of contention [28]. Of particular relevance to the study we present here, analysis of blood and urine of rats fed a diet supplemented with Roundup-tolerant NK603 GM corn for 90 days showed statistically significant differences in multiple components [29], which has been suggested may constitute early signs of liver and kidney toxicity [30]. In a follow-up chronic toxicity, investigation rats were fed the same NK603 GM corn for a 2-year period in order to determine if the statistically significant differences in urine and blood biochemistry, which could be interpreted as signs of liver and kidney dysfunction, did indeed escalate into serious disease. The results obtained included blood/urine biochemical changes indicative of liver and kidney structure and functional pathology [31].

In an effort to obtain deeper insight into these findings that could be taken as signs of kidney and liver pathology, we conducted a full transcriptomic and metabolomic analysis of these organs from the female cohort of animals fed a diet supplemented with 33% NK603 GM corn either with or without Roundup application during cultivation. Overall, although statistically significant differences in several metabolites that were indicative of organ damage were observed in the metabolomics analysis between test and control animals fed the non-GM isogenic equivalent corn, high false discovery rates prevented definitive conclusions of harm or safety. In addition, differences observed between individuals within a given group were greater than the metabolic effect of the different diets. Even if the biological relevance of the statistically significant differences presented in this study is unclear, we make our results available for comparison in future studies investigating the potential toxicological properties of the NK603 corn.

## Methods

### Experimental design

The tissues analysed in this study were obtained from animals as previously described [31]. Briefly, the experimental protocol is as follows. The varieties of maize used in this study were DKC 2678 Roundup-tolerant NK603 (Monsanto Corp., USA) and its nearest isogenic non-transgenic control DKC 2675. Harlan Sprague–Dawley rats at 5 weeks of age were randomly assigned on a weight basis into groups of 10 animals. For each sex, one control group had access to plain water and standard diet from the closest isogenic non-transgenic maize control; six groups were fed with 11, 22 and 33% of GM NK603 maize either treated or not with Roundup at 3 L/ha (WeatherMAX, 540 g/L of glyphosate, EPA Reg. 524–537). Clinical and biochemical parameters measured have been extensively described [31]. Animals were sacrificed at the same time of day during the course of the study either to comply with animal welfare regulations to avoid unnecessary suffering or at the termination of the study period of 2 years. Liver and kidneys were divided in two and one half snap-frozen in liquid nitrogen/dry ice and stored at −80 °C.

### Transcriptome analysis

Transverse cross-sectional slices of liver and kidneys were processed for total RNA extraction using MagMax-96 for Microarrays Total RNA Isolation Kit (Ambion, Life Technologies Ltd, Paisley, UK). Total RNA (500 ng) was labelled using terminal deoxynucleotidyl transferase (TdT) in the presence of a proprietary biotinylated compound using the Ambion whole transcript Expression kit and the whole transcript Terminal Labelling kit (Affymetrix UK Ltd., High Wycombe, UK), following the standard protocols. We employed the Affymetrix GeneChip^®^ Rat Gene 2.0 ST Array containing approximately 610,400 probes grouped into 214,300 exon-level and 26,400 gene-level probe sets. Hybridization cocktails were applied to Affymetrix Rat Gene 2.0 microarrays and processed in accordance with the manufacturer’s recommended procedure using the GCS3000 microarray system (Affymetrix). Array data were exported as cell intensity (CEL) files for further analysis. CEL files were normalized together in the Expression Console software package (Affymetrix), using the Robust Multi-array Average (RMA) sketch algorithm (gene-level). Data were quality control assessed by using standard metrics and guidelines for the Affymetrix microarray system. Normalized data files (CHP files) were imported into Omics Explorer 3.0 (Qlucore) for further quality control and statistical analysis. Data used for the functional analysis were selected at the statistical cut-off values of *p* < 0.01 with FC >1.1 [32]. The pathway analysis was done using the Thomson Reuters MetaCore Analytical Suite and/or the NIH Database for Annotation, Visualization and Integrated Discovery Bioinformatics Resources 6.7 (DAVID) using recommended analytical parameters [32]. These microarray data have been submitted to Gene Omnibus and are accessible through accession number GSE73888.

### Metabolome analysis

Semi-quantitative metabolomics analysis was performed by ultrahigh performance liquid chromatography-tandem mass spectroscopy (UPLC-MS/MS) and gas chromatography-mass spectroscopy (GC–MS) at Metabolon Inc. (Durham, NC, USA) as previously described [33, 34]. Briefly, samples prepared using Metabolon’s standard extraction were divided into five fractions: one for analysis by UPLC-MS/MS with positive ion mode electrospray ionization, one for analysis by UPLC-MS/MS with negative ion mode electrospray ionization, one for LC polar platform, one for analysis by GC–MS, and one sample was reserved for backup. A quality control value assessment was undertaken to determine instrument variability by calculating the median relative standard deviation (RSD) for the internal standards that were pre-mixed into each sample prior to injection into the mass spectrometer. This yielded a value of 6% for instrument variability. Overall process variability as determined by calculating the median RSD for all endogenous metabolites (that is, non-instrument standards) present in 100% of the samples gave a value of 11–12%.

Raw data were extracted, peak-identified and QC processed using Metabolon’s hardware and software [35]. Metabolites were identified by automated comparison and curated by visual inspection for quality control using software developed at Metabolon [36]. Peaks were quantified using area under the curve. The maximum percent missing data allowed was 20%. As a result, 647 and 593 metabolites were taken forward for bioanalytical analysis in kidney and liver tissues, respectively. The language and statistical environment R was employed in order to explore the relationship between the control and the treated samples. We regressed out the batch effects to correct variation resulting from instrument inter-day tuning differences using the limma package removeBatchEffect [37]. Pairwise non-parametric Mann–Whitney U tests were performed and a *p* value was attributed to each of the metabolites. The resulting *p* values were adjusted by the Benjamini–Hochberg multi-test adjustment method for a high number of comparisons. Volcano plots were also constructed in order to visualize the differences in metabolite and protein expression for each of the comparisons. The aforementioned tests and plots were performed using in-house R scripts.

## Results

### Tissue selection

Rat liver and kidney tissues were obtained from animals that formed part of a chronic (2-year) feeding study looking at potential toxic effects arising from the consumption of the Roundup-tolerant GM maize NK603. The three groups of animals that formed the focus of this investigated were fed standard laboratory rat chow diets supplemented with 33% NK603 GM maize (NK603-R), 33% NK603 GM maize plus Roundup application during cultivation (NK603+R) and a control diet with 33% non-GM isogenic maize. Most male rats were discovered after death had occurred. This resulted in organ necrosis making them unsuitable for further analysis. We therefore focused our investigation on female animals where freshly dissected tissues from cohorts of 9–10 euthanized treated and untreated rats were available. Female controls were euthanized at 701 ± 62 days. Rats fed NK603-R and NK603+R were, respectively, euthanized at 618 ± 148 and 677 ± 83 days. Female animals mostly died from mammary tumours (8 on 5 controls rats, 15 on 8 NK603-R rats, and 13 on 9 NK603+R rats). The objective of this investigation was to obtain deeper insight into the biology of the liver and kidneys from this cohort of female animals by a molecular profiling (transcriptomics, metabolomics) analytical approach.

### Transcriptomics analysis

**Fig. 1:**
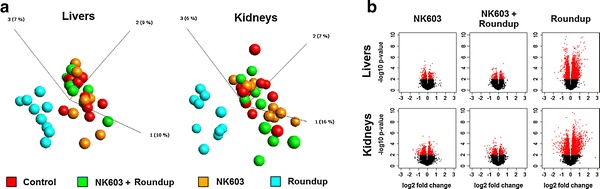
Wide-scale transcriptome profiles in liver and kidneys of NK603-fed rats. Liver and kidneys from control rats and animals fed NK603 GM maize either with or without Roundup application during the cultivation cycle were subjected to a full microarray transcriptome analysis. **a** PCA analysis of transcript cluster expression profiles shows no distinct separation into groups of treated (*orange* and *green*) and control (*red*) rats in both kidney and liver samples. By comparison, rats administered with Roundup (*blue*) in drinking water from the same experiment and subjected to the same transcriptome analysis clearly separate from the control and NK603 maize-fed groups. Each *sphere* represents the result of a single animal. **b** Volcano plots of the liver and kidney transcriptome profiles. Transcript cluster expression derived from the transcriptome profile data of liver and kidneys of control and NK603 maize-fed groups, either with or without Roundup (R) application, was plotted as log 2 fold change against −log10 *p* values. Each *dot* represents a single transcript cluster

**Fig. 2:**
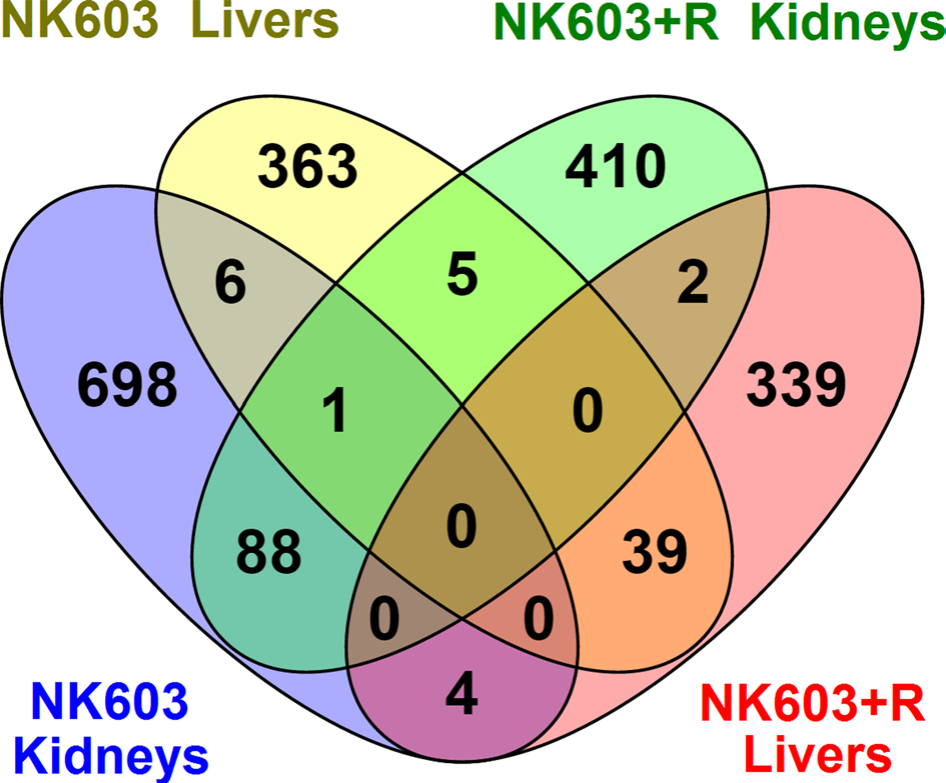
Venn diagram showing numbers of genes commonly disturbed in liver and kidney. Data were selected at *p* < 0.01 and fold changes >1.1

**Fig. 3:**
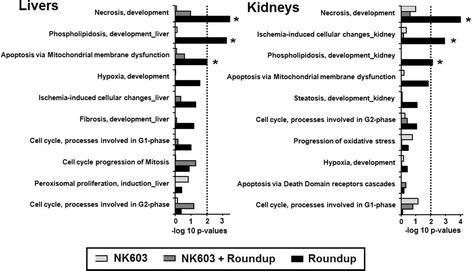
Toxicity ontology analysis of genes disturbed in liver and kidneys of NK603 fed rats. List of toxicity process networks as revealed by MetaCore analysis of transcriptome profiles of liver and kidney from female rats fed NK603 GM maize either with or without Roundup application during the cultivation cycle or receiving 0.1 ppb of Roundup (50 ng/L glyphosate) in drinking water (*p* < 0.01, fold changes >1.1). The *p* values are determined by hypergeometric calculation

The transcriptome dataset obtained via microarray analysis was initially subjected to an unsupervised Principal Component Analysis (PCA). This analysis reduces a high-dimensional expression profile to single variables (components) retaining most of the variation. The distribution of the samples in a 3D space defined by three PCA components allows an estimation of the effects of the treatment or the detection of outliers. The results (Fig. 1a) showed no segregation of the GM NK603 corn-fed groups from the control animals, indicating that the treatment was not a major source of difference. In contrast, rats administered via drinking water with 0.1 ppb Roundup (50 ng/L glyphosate equivalent concentration) were clearly separated in this PCA analysis from the controls and NK603 corn-fed groups (Fig. 1) as previously reported [38]. Figure 1b shows the statistical significance (by Student’s *t* tests) of differential transcript cluster expression in a volcano plot format along with respective fold changes (FC). This allows a visualization of the distribution of any statistically significant differences. Overall, although some significant statistical differences were measured, false discovery rates ranged from 43 to 83% at the chosen cut-off *p* value of 1% (Table 1). Statistical analysis simulating random samples confirms that the degree of statistical difference between control and GM NK603 corn treatment groups can arise by chance (Table 1). A Venn diagram comparing liver and kidney transcript cluster expression profiles at these thresholds (Fig. 2) indicates that most of the statistical differences were tissue-specific. Indeed, there was no gene having its expression disturbed by the NK603±R in both liver and kidneys. Even if the level of statistical significance does not survive the multiple comparison tests, biological interpretation could provide coherent explanations of the treatment effect if these statistically significant differences were concentrated in pathways reflective of a disease state of these organs. Thus, we conducted a functional disturbances analysis with the Thomson Reuters MetaCore Analytical Suite (Fig. 3). Results obtained for rats administered via drinking water with 0.1 ppb Roundup clearly shows alterations in the transcriptome profile (apoptosis, necrosis, phospholipidosis, mitochondrial membrane dysfunction and ischemia) correlating with the observed increased signs of anatomical and functional pathology of the liver and kidneys [38]. By contrast, alterations in gene expression provoked by NK603 corn treatment were not reflective of liver and kidney toxic effects (Fig. 3).

**Table 1:** Number of transcript clusters whose expression is disturbed at different cut-off threshold *p* values

*p* value	Liver NK603+R	Kidneys NK603+R	Liver NK603	Kidneys NK603	Random	Liver Roundup	Kidneys Roundup
0.05	2126^(0.83)^	2337^(0.78)^	2119^(0.86)^	3176^(0.58)^	1835^(0.98)^	8606^(0.21)^	8656^(0.21)^
0.01	393^(0.83)^	543^(0.67)^	425^(0.83)^	838^(0.43)^	380^(0.96)^	4224^(0.08)^	4447^(0.08)^
0.001	29^(0.83)^	58^(0.58)^	54^(0.58)^	127^(0.29)^	31^(0.95)^	1593^(0.02)^	1894^(0.02)^
0.0001	1^(0.61)^	8^(0.40)^	7^(0.77)^	14^(0.25)^	1^(0.95)^	630^(0.006)^	764^(0.005)^
0.00001	0	1^(0.15)^	1^(0.24)^	2^(0.11)^	0	230^(0.002)^	219^(0.002)^

Transcriptomics results of liver and kidneys from rats fed NK603 GM maize either with (NK603+R) or without (NK603) Roundup application during the cultivation cycle summarizing the number of genes whose expression was altered at different *p* value thresholds. The number in superscript parenthesis is the maximal *q* value (calculated using Benjamini–Hochberg method according to corresponding to the number of genes found disturbed at increasing (0.05 to 0.00001) *p* value stringency. A statistical analysis of simulated random samples was also performed to estimate effects that would be expected to arise by chance. The number of genes disturbed by the Roundup treatment in the same experiment is given for comparison

### Metabolomics analysis

Although transcriptome analyses reveal alterations in gene function that could be correlated with toxicological processes, they do not always translate into metabolic disturbances. Thus, in order to obtain insight into organ damage, we next conducted a metabolome profiling of liver and kidney sections of GM NK603 (±Roundup) corn treatment groups from the same animals to ascertain any changes in metabolites that could be indicative of disease. In this study, 692 and 673 metabolites were, respectively, identified in kidney and liver tissues. In order to ascertain if Roundup residues were bioaccumulating, we also measured the presence of glyphosate (*N*-(phosphonomethyl) glycine) and its metabolite aminomethylphosphonic acid (AMPA). At a limit of detection of 7.8 ppb, neither glyphosate nor AMPA was found to be present in these tissues.

**Fig. 4:**
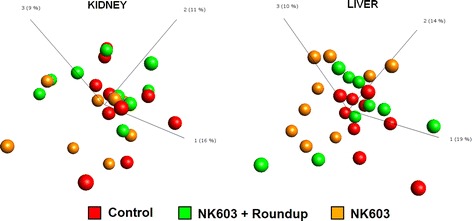
PCA analysis of metabolome profiles of liver and kidneys. Liver and kidneys from control rats and animals fed NK603 GM maize either with or without Roundup application during the cultivation cycle were subjected to a metabolome analysis. PCA analysis of metabolite levels show no distinct separation into groups of treated (*orange* and *green*) and control (*red*) rats in both kidney and liver samples. Each *sphere* represents the result of a single animal

**Fig. 5:**
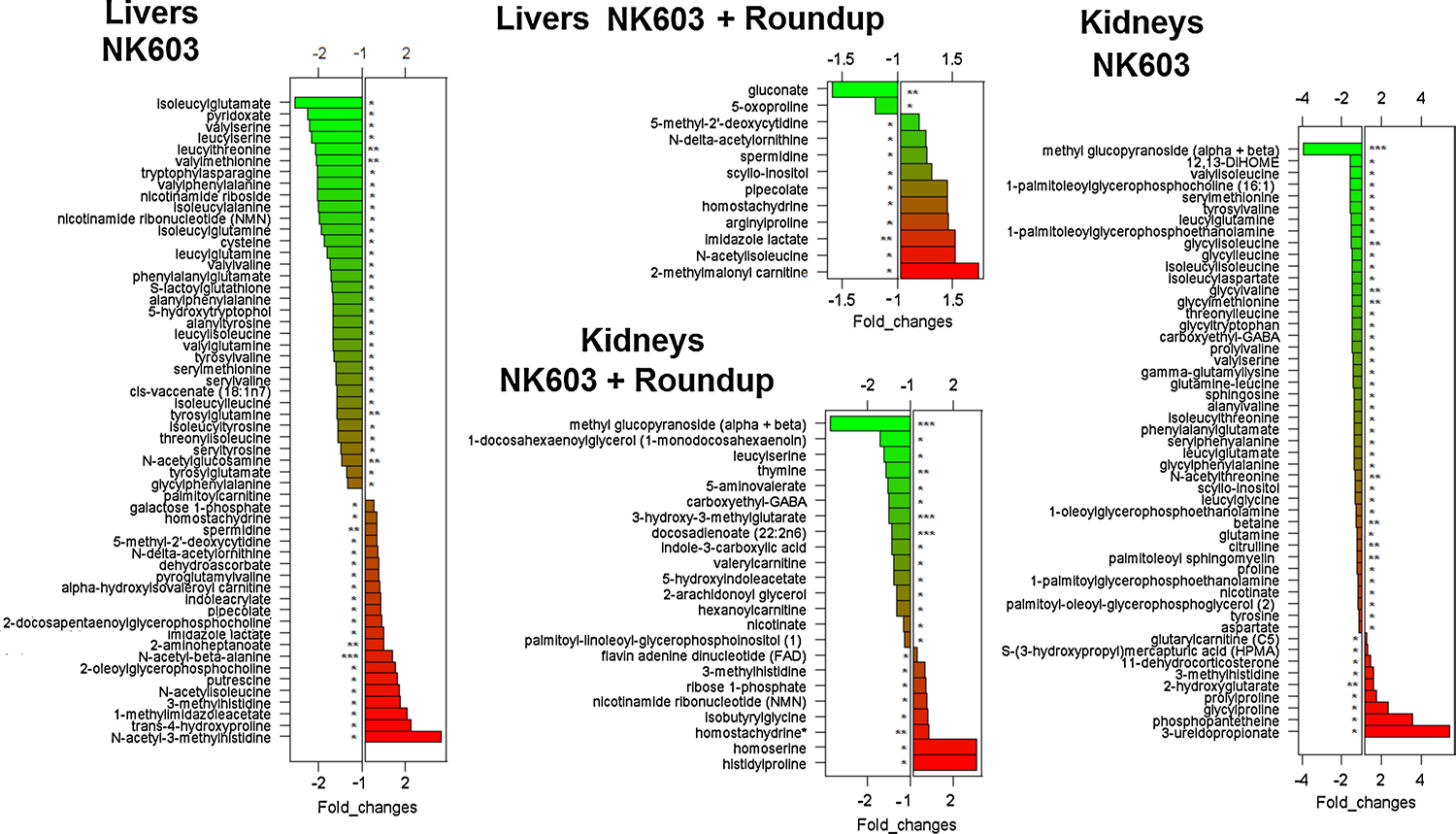
Score plots of the significantly altered metabolites in liver and kidneys of NK603-fed rats. Levels of each metabolite from the metabolomics of liver and kidneys from female rats fed NK603 GM maize either with (NK603+R) or without (NK603) Roundup application during the cultivation cycle were subjected to a statistical analysis by comparison to controls using a Mann–Whitney U test. All metabolites showing a statistically significant change are shown. *p < 0.05; **p < 0.01, ***p < 0.001

Similarly to what was observed in the transcriptomics analysis (Fig. 1), samples of neither kidney nor liver from a particular test group clustered together in a PCA analysis (Fig. 4) with all the raw data available in Additional file 1: Table S1. Additionally, none of the statistically significant disturbances detected survived a false discovery rate analysis. Statistically significant changes were observed in the levels of some metabolites in test groups compared to controls; 23 in NK603+R kidneys, 51 in NK603 kidneys, 12 in NK603+R livers and 56 in NK603 livers (Fig. 5). No toxicologically relevant functional disturbances were found following analysis performed with the Thomson Reuters MetaCore Analytical Suite. A statistically significant increase in 3-methylhistidine levels was noticed in the kidneys of both NK603±R-fed groups (Fig. 5). Only 4 compounds, namely methyl glucopyranoside, carboxyethyl-GABA, 3-methylhistidine, nicotinate, were commonly altered after NK603 or NK603+Roundup treatments in kidneys. Additionally, the level of many dipeptides was found to be lower relative to controls in the kidney as well as in the liver of animals fed NK603±R. Most dipeptides showing an alteration in their level included those with a branched-chain amino acid. By contrast, proline-containing dipeptides (glycylproline, arginylproline, histidylproline) were significantly increased.

**Fig. 6:**
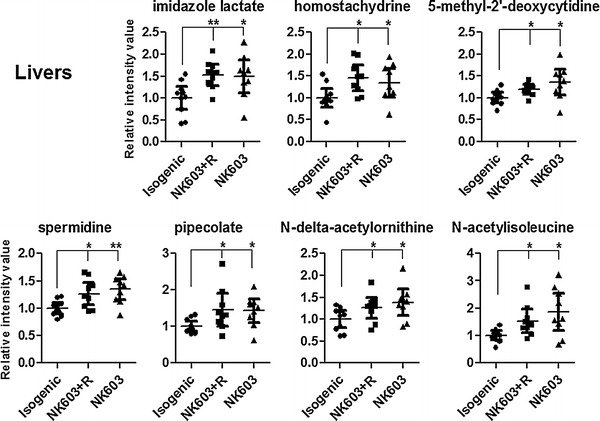
Scatter plots of the major significantly altered metabolites in liver of rats fed NK603 GM maize. Levels of the 7 metabolites commonly deregulated in livers from rats fed NK603 GM maize either with (NK603+R) or without (NK603) Roundup application during the cultivation cycle are shown. *p < 0.05; **p < 0.01

In livers, a total of 7 metabolites, namely 5-methyl-2′-deoxycytidine, *N*-delta-acetylornithine, spermidine, pipecolate, homostachydrine, imidazole lactate, *N*-acetylisoleucine, were commonly disturbed in animals treated with NK603 or NK603+Roundup and could be indicative of an effect of this GM maize. Among them, pipecolate and its analogue homostachydrine had their levels elevated. Interestingly, homostachydrine is a phytochemical and it is likely that this compound originated from the NK603 diet itself rather than from endogenous metabolism. The levels of putrescine, spermidine and the spermidine degradation product Acisoga (*N*-(3-Acetamidopropyl) pyrrolidin-2-one) were found to be elevated (Fig. 5). However, the trend was not always statistically significant. Individual variation of these 7 metabolites was also investigated (Fig. 6). The distribution of normalized intensity values between control and test groups considerably overlapped. Thus, differences between individual animals within a group were greater than the effect of test diets (Fig. 6).

## Discussion

We report here the first in vivo transcriptomics and metabolomics analysis in an established rat toxicity model system following long-term (2 year) consumption of an agricultural GM food, namely NK603 Roundup-tolerant maize.

The metabolite 3-methylhistidine, which was found elevated in the kidney tissue of animals fed NK603 (Fig. 5), is an indicator of protein catabolism. The dipeptide 3-methylhistidine is formed by the posttranslational methylation of histidine in the contractile proteins of skeletal muscle, actin and myosin. Proteolytic degradation of muscle tissues generates free 3-methylhistidine, which is slowly released into the bloodstream, where it is efficiently filtered by the kidney. The measurement of 3-methylhistidine provides an index of the rate of muscle protein breakdown [39]. In a previous investigation of the kidney metabolome, one of the most significant effects induced by three nephrotoxins was a decrease in dipeptides [40]. In liver, we observed an accumulation of polyamines (putrescine and spermidine), which could suggest an elevated metabolic state (Fig. 5). Typically, polyamines are highly active in rapidly proliferating cells, although their levels fluctuate during the cell cycle and accumulate in regenerating liver [41]. The increased levels of polyamines in liver could also be explained by an increased dietary intake since the NK603 maize tested here has been found to contain more polyamines than its near-isogenic counterpart [42]. However, the biological relevance of the metabolic alterations observed in this study remains to be ascertained.

Residues of the GBH were neither detected in liver and kidneys of animals fed NK603 maize sprayed with Roundup during cultivation at a level of detection of 7.8 ppb, nor in the grain itself (level of quantification 10 ppb) (Mesnage and Antoniou, unpublished results). This in all likelihood is due to the fact that Roundup was applied early during the cultivation cycle. This also provides an explanation as to why we observed no difference in either the transcriptome or metabolome profiles between the two test and the control groups. The presence of GBH residues in the feed would have been expected to show statistically significant differences in these molecular profiling parameters since consumption of an ultra-low dose of Roundup (4 ng/kg body weight/day glyphosate equivalent exposure) administered via drinking water showed marked, highly significant differences in the transcriptome of both liver and kidneys of rats, which was reflective of structure and functional damage in these organs [31]. More recently, we have shown that proteome and metabolome profiles from the same cohort of female animals reveal the presence of non-alcoholic fatty liver disease [43]. It should be noted that a single application of GBH during cultivation of glyphosate-tolerant GM crops as conducted in the generation of the NK603 maize grain used in this study is not a typical farming practice. Generally, at least two applications of GBH are employed, early and late during the cultivation cycle, resulting in residues of glyphosate and AMPA being readily detectable in the harvested crop [7].

## Conclusions

In conclusion, the analyses presented here for liver and kidneys from rats fed a diet containing 33% NK603 GM maize were not sufficient to definitively conclude on the molecular effects underlying the pathologies previously reported in these organs [31]. Therefore, it is not possible to arrive at conclusion of either harm or safety from the consumption of this GM maize. Although our results are largely negative with respect to differences between test and control groups, they are nevertheless informative and thus we make them available for comparison in future studies investigating the toxicological properties of the NK603 corn.

## Additional file

**Additional file 1: Table S1.** Raw Data of the metabolome analysis. Raw peak intensity values are indicated for each of the 692 and 673 metabolites respectively identified in  kidney and liver tissues.

## References

[1] Van Montagu M (2011). It is a long way to GM agriculture. Annu Rev Plant Biol.

[2] Nicolia A, Manzo A, Veronesi F, Rosellini D (2014). An overview of the last 10 years of genetically engineered crop safety research. Crit Rev Biotechnol.

[3] Van Eenennaam AL, Young AE (2014). Prevalence and impacts of genetically engineered feedstuffs on livestock populations. J Anim Sci.

[4] Hilbeck A, Binimelis R, Defarge N, Steinbrecher R, Székács A, Wickson F, Antoniou M, Bereano PL, Clark EA, Hansen M, Novotny E, Heinemann J, Meyer H, Shiva V, Wynne B (2015). No scientific consensus on GMO safety. Environ Sci Eur.

[5] James C (2015) Global Status of Commercialized Biotech/GM Crops: 2015. ISAAA Brief 51

[6] Benbrook CM (2016). Trends in glyphosate herbicide use in the United States and globally. Environ Sci Eur.

[7] Bohn T, Cuhra M, Traavik T, Sanden M, Fagan J, Primicerio R (2014). Compositional differences in soybeans on the market: glyphosate accumulates in Roundup Ready GM soybeans. Food Chem.

[8] Mesnage R, Defarge N, Rocque LM, Spiroux de Vendomois J, Seralini GE (2015). Laboratory rodent diets contain toxic levels of environmental contaminants: implications for regulatory tests. PLoS ONE.

[9] Mesnage R, Defarge N, Spiroux de Vendomois J, Seralini GE (2015). Potential toxic effects of glyphosate and its commercial formulations below regulatory limits. Food Chem Toxicol.

[10] Antoniou M, Habib MEM, Howard CV, Jennings RC, Leifert C, Nodari RO (2012). Teratogenic effects of glyphosate-based herbicides: divergence of regulatory decisions from scientific evidence. J Environ Anal Toxicol.

[11] Krüger M, Schrödl W, Neuhaus J, Shehata A (2013). Field investigations of glyphosate in urine of Danish dairy cows. J Environ Anal Toxicol.

[12] Guyton KZ, Loomis D, Grosse Y, El Ghissassi F, Benbrahim-Tallaa L, Guha N, Scoccianti C, Mattock H, Straif K (2015). Carcinogenicity of tetrachlorvinphos, parathion, malathion, diazinon, and glyphosate. Lancet Oncol.

[13] Myers JP, Antoniou MN, Blumberg B, Carroll L, Colborn T, Everett LG, Hansen M, Landrigan PJ, Lanphear BP, Mesnage R, Vandenberg LN, vom Saal FS, Welshons WV, Benbrook CM (2016). Concerns over use of glyphosate-based herbicides and risks associated with exposures: a consensus statement. Environ Health.

[14] Fonseca C, Planchon S, Serra T, Chander S, Saibo NJ, Renaut J, Oliveira MM, Batista R (2015). In vitro culture may be the major contributing factor for transgenic versus nontransgenic proteomic plant differences. Proteomics.

[15] Latham JR, Wilson AK, Steinbrecher RA (2006). The mutational consequences of plant transformation. J Biomed Biotechnol.

[16] Wilson AK, Latham JR, Steinbrecher RA (2006). Transformation-induced mutations in transgenic plants: analysis and biosafety implications. Biotechnol Genet Eng Rev.

[17] Rang A, Linke B, Jansen B (2005). Detection of RNA variants transcribed from the transgene in Roundup Ready soybean. Eur Food Res Technol.

[18] Herrero M, Ibanez E, Martin-Alvarez PJ, Cifuentes A (2007). Analysis of chiral amino acids in conventional and transgenic maize. Anal Chem.

[19] Manetti C, Bianchetti C, Casciani L, Castro C, Di Cocco ME, Miccheli A, Motto M, Conti F (2006). A metabonomic study of transgenic maize (*Zea mays*) seeds revealed variations in osmolytes and branched amino acids. J Exp Bot.

[20] Zolla L, Rinalducci S, Antonioli P, Righetti PG (2008). Proteomics as a complementary tool for identifying unintended side effects occurring in transgenic maize seeds as a result of genetic modifications. J Proteome Res.

[21] Shepherd LV, Hackett CA, Alexander CJ, McNicol JW, Sungurtas JA, Stewart D, McCue KF, Belknap WR, Davies HV (2015). Modifying glycoalkaloid content in transgenic potato—metabolome impacts. Food Chem.

[22] Jiao Z, Si XX, Li GK, Zhang ZM, Xu XP (2010). Unintended compositional changes in transgenic rice seeds (*Oryza sativa* L.) studied by spectral and chromatographic analysis coupled with chemometrics methods. J Agric Food Chem.

[23] Zhou J, Ma C, Xu H, Yuan K, Lu X, Zhu Z, Wu Y, Xu G (2009). Metabolic profiling of transgenic rice with cryIAc and sck genes: an evaluation of unintended effects at metabolic level by using GC-FID and GC-MS. J Chromatogr B Analyt Technol Biomed Life Sci.

[24] Li X, Ding C, Wang X, Liu B (2015). Comparison of the physiological characteristics of transgenic insect-resistant cotton and conventional lines. Scientific Rep.

[25] Agapito-Tenfen SZ, Vilperte V, Benevenuto RF, Rover CM, Traavik TI, Nodari RO (2014). Effect of stacking insecticidal cry and herbicide tolerance epsps transgenes on transgenic maize proteome. BMC Plant Biol.

[26] Agapito-Tenfen SZ, Guerra MP, Wikmark OG, Nodari RO (2013). Comparative proteomic analysis of genetically modified maize grown under different agroecosystems conditions in Brazil. Proteome Sci.

[27] Dona A, Arvanitoyannis IS (2009). Health risks of genetically modified foods. Crit Rev Food Sci Nutr.

[28] Spiroux de Vendômois J, Cellier D, Velot C, Clair E, Mesnage R, Seralini GE (2010). Debate on GMOs health risks after statistical findings in regulatory tests. Int J Biol Sci.

[29] Hammond B, Dudek R, Lemen J, Nemeth M (2004). Results of a 13 week safety assurance study with rats fed grain from glyphosate tolerant corn. Food Chem Toxicol.

[30] Spiroux de Vendômois J, Roullier F, Cellier D, Seralini GE (2009). A comparison of the effects of three GM corn varieties on mammalian health. Int J Biol Sci.

[31] Seralini GE, Clair E, Mesnage R, Gress S, Defarge N, Malatesta M (2014). Republished study: long-term toxicity of a Roundup herbicide and a Roundup-tolerant genetically modified maize. Environ Sci Eur.

[32] Huang W, Sherman BT, Lempicki RA (2009). Systematic and integrative analysis of large gene lists using DAVID bioinformatics resources. Nat Protoc.

[33] Evans AMBB, Liu Q, Mitchell MW, Robinson RJ (2014). High resolution mass spectrometry improves data quantity and quality as compared to unit mass resolution mass spectrometry in high-throughput profiling metabolomics. Metabolomics.

[34] Ohta T, Masutomi N, Tsutsui N, Sakairi T, Mitchell M, Milburn MV, Ryals JA, Beebe KD, Guo L (2009). Untargeted metabolomic profiling as an evaluative tool of fenofibrate-induced toxicology in Fischer 344 male rats. Toxicol Pathol.

[35] Dehaven Cd EA DH, Lawton Ka (2012) Software techniques for enabling high-throughput analysis of metabolomic datasets. In: Roessner U (ed) Metabolomics. InTech. ISBN:978-953-51-0046-1. doi:10.5772/31277. http://www.intechopen.com/books/metabolomics/software-techniques-for-enabling-high-throughput-analysis-on-metabolomic-datasets

[36] Dehaven CD, Evans AM, Dai H, Lawton KA (2010). Organization of GC/MS and LC/MS metabolomics data into chemical libraries. J Cheminf.

[37] Ritchie ME, Phipson B, Wu D, Hu Y, Law CW, Shi W, Smyth GK (2015). limma powers differential expression analyses for RNA-sequencing and microarray studies. Nucleic Acids Res.

[38] Mesnage R, Arno M, Costanzo M, Malatesta M, Séralini G-E, Antoniou MN (2015). Transcriptome profile analysis reflects rat liver and kidney damage following chronic ultra-low dose Roundup exposure. Environ Health.

[39] Aranibar N, Vassallo JD, Rathmacher J, Stryker S, Zhang Y, Dai J, Janovitz EB, Robertson D, Reily M, Lowe-Krentz L, Lehman-McKeeman L (2011). Identification of 1- and 3-methylhistidine as biomarkers of skeletal muscle toxicity by nuclear magnetic resonance-based metabolic profiling. Anal Biochem.

[40] Boudonck KJ, Mitchell MW, Nemet L, Keresztes L, Nyska A, Shinar D, Rosenstock M (2009). Discovery of metabolomics biomarkers for early detection of nephrotoxicity. Toxicol Pathol.

[41] Ogiso S, Matsumoto T, Nimura Y (1997). The role of polyamines in liver regeneration after hepatectomy with ischemic injury. Surg Today.

[42] Mesnage R, Agapito-Tenfen S, Vilperte V, Renney G, Ward M, Séralini GE, Antoniou M (2016). An integrated multi-omics analysis of the NK603 Roundup-tolerant GM maize reveals metabolism disturbances caused by the transformation process. Sci Rep.

[43] Mesnage R, Renney G, Séralini GE, Ward M, Antoniou M (2017). Multiomics reveal non-alcoholic fatty liver disease in rats following chronic exposure to an ultra-low dose of Roundup herbicide. Sci Rep.

